# The Inhibitory Effect of Regulatory T Cells on the Intimal Hyperplasia of Tissue-Engineered Blood Vessels in Diabetic Pigs

**DOI:** 10.3389/fbioe.2022.929867

**Published:** 2022-07-26

**Authors:** Fengjie Guo, Zhipeng Ren, Dongxu Liu, Linghui Wang, Xiaobin Hou, Wen Chen

**Affiliations:** ^1^ Outpatient Department, The 8th Medical Center, Chinese PLA General Hospital, Beijing, China; ^2^ Department of Thoracic Surgery, The First Medical Center, Chinese PLA General Hospital, Beijing, China; ^3^ Department of Pathology, The 8th Medical Center, Chinese PLA General Hospital, Beijing, China

**Keywords:** regulatory T cell, diabetes, tissue-engineered blood vessel, endothelial progenitor cell, intimal hyperplasia

## Abstract

Severe inflammatory response and functional impairment of endothelial progenitor cells (EPCs) often lead to the implantation failure of EPC-captured tissue-engineered blood vessels (TEBVs) in diabetes. Regulatory T cells (Treg cells) are the most important inhibitory immune cells, but their effects in angiogenesis remain undefined, and the differences in the microenvironment may be an important reason. Here, we constructed a TEBV coated with an anti-CD34 antibody-functionalized heparin-collagen multilayer (anti-CD34 antibody-modified TEBV) using layer-by-layer self-assembly. Then, TEBVs were implanted into diabetic pigs. All TEBVs remained unobstructed 60 days after implantation, although varying degrees of intimal hyperplasia were detectable. Severe intimal hyperplasia was observed in the control group and peripheral injection of Treg cells group. Intravenous injection of Treg cells significantly inhibited intimal hyperplasia, inflammation, and cell apoptosis. Moreover, intravenous injection increased the proportion of circulating EPCs, while peripheral injection did not have these effects and reduced microvessel density around the TEBV. Interestingly, many Nestin^+^ cells could be detected in TEBVs, most of which were fusiform, showing the characteristics of smooth-muscle cells. Treg cell intravenous transplantation markedly reduced the number of Nestin^+^ cells in the TEBV. In conclusion, Treg cells inhibited the intimal hyperplasia of TEBVs in diabetic pigs by promoting EPC mobilization, anti-inflammatory action, and cellular protection.

## Introduction

In recent years, the incidence and mortality of cardiovascular and cerebrovascular diseases have increased year by year, leading to a significant increase in the clinical demand for tissue-engineered blood vessels (TEBVs) (Song et al., 2018). Endothelial progenitor cells (EPCs) were first reported in the late 20th century and specifically express CD34, CD133, VEGFR-2 (KDR), and CD144 (Afshari et al., 2020; Naserian et al., 2020; Xing et al., 2020; Nouri Barkestani et al., 2021). Many scientists constructed EPC-captured TEBVs using CD34 or CD133 antibodies, and animal experiments showed that these TEBVs can achieve rapid endothelialization (Rotmans et al., 2005). However, most patients who need TEBV implantation clinically have underlying diseases such as diabetes. Our previous study showed that the long-term patency rate of EPC-captured TEBV in diabetic rats was very low, which may be due to various reasons (Chen et al., 2015b). High glucose reduced the number and function of homing EPCs and induced the pathological proliferation of vascular smooth muscle cells (Chen et al., 2015a). Therefore, it is of great clinical significance to improve the patency rate of TEBV in diabetes mellitus.

TEBV implantation inevitably activates the recipient’s immune system, and excessive inflammatory response can impair homing EPC function. The occurrence and development of diabetes mellitus are closely related to chronic inflammation and abnormal immune regulation (Halim and Halim, 2019). High glucose can cause excessive activation of the inflammatory response, further damage migrating endothelial cells and homing EPCs, and may cause implantation failure of EPC-captured TEBV (Petrelli et al., 2012). Regulatory T cells (Treg cells) are the primary inhibitory immune cells that can down-regulate an overactive immune response by secreting immunosuppressive molecules or by direct cellular contact (Kawada, 2018). In diabetic patients, Treg cell numbers were significantly decreased, while Th1 cell proliferation and interferon-γ concentrations were increased, leading to excessive inflammatory responses (Qin et al., 2021; Zhou et al., 2021). We speculate that Treg cell transplantation may be an effective method to improve the patency of TEBV in diabetes mellitus.

The interaction between Treg cells and angiogenesis was considered to be “a dark double track” (Gasparri et al., 2013). Some studies have found that Treg cells are an important source of VEGF in cancer, and the elimination of Treg cells can significantly reduce the production of VEGF. However, other studies have shown that Treg cells inhibit angiogenesis in ischemic tissue by reducing T cell and macrophage infiltration (Zouggari et al., 2009). Many studies have also reported the association between Treg cells and EPCs, and the proportion of Treg cells in peripheral blood is positively correlated with the proportion of early EPCs (Choi et al., 2018). These results suggest that the microenvironment may be an important factor affecting the regulation of angiogenesis by Treg cells. After TEBV implantation, the peripheral blood circulating in the TEBV contains sufficient oxygen and nutrients, while the damaged tissues around TEBV are relatively ischemic and hypoxia, so it can be used as an important model to study the relationship between Treg cells and angiogenesis. Our preliminary results showed that the vascular wall thickness of mice, rats, and rabbits was very thin, and oxygen and nutrients can permeate the whole layer of TEBV. In this study, we constructed a diabetic pig model to study the effect of Treg cells on EPC-captured TEBV in diabetes and to explore the relationship between Treg cells and angiogenesis.

## Materials and Methods

### Construction of an Anti-CD34 Antibody-Modified TEBV

The carotid arteries of female Landrace pigs weighing about 60 kg were used. The blood was repeatedly rinsed with normal saline, and the surrounding fat and connective tissue were removed. The blood vessels were placed in 0.5% trypsin (Hyclone) and incubated at 37° for 12 h. After washing with normal saline three times, the vessels were placed in 0.5% TritonX-100 hypotonic solution (0.01 mol/L TrIS-HCl), shaken, and washed for 24 h. The vessels were then placed in 0.5% TritonX-100 hypertonic solution (1.5 mol/L KCL solution), shaken, and washed for 24 h. After full washing of normal saline, an acellular vascular scaffold was obtained.

We constructed TEBV coated with an anti-CD34 antibody functionalized heparin-collagen multilayer (anti-CD34 antibody modified TEBV) using layer-by-layer self-assembly. The acellular vascular scaffold was immersed in 3 mg/ml PEI solution (Sigma) for 2 h. After washing with PBS three times, immersing vascular scaffold in 1 mg/ml heparin solution (negative charge) for 15 min. After washing with ABS buffer three times, the vascular scaffold was immersed in 1 mg/ml collagen solution (positive charge) for 15 min. Heparin solution and collagen solution were alternately treated 10 times to obtain a vascular scaffold coated with a heparin-collagen multilayer. After washing with ABS buffer three times, the vascular scaffold was placed in 0.25% glutaraldehyde for 2 h and washed with PBS 3 times. Then placing it in 60 ug/mL anti-CD34 antibody solution (Abcam) and incubated for 12 h under dark conditions. After washing with PBS three times, anti-CD34 antibody modified TEBV was obtained.

### Hemolytic Test

The experiment was divided into five groups: negative control group (0.9% NaCl solution), positive control group (Double distilled water), matrix group (Acellular vascular matrix), Hep-Col group (TEBV scaffold after coating 10 layers), and CD34 group (anti-CD34 antibody modified TEBV). 4 ml of anticoagulant peripheral blood from pigs was collected and then diluted with 0.9% NaCl solution in a ratio of 4:5. Then diluted blood was added to each group, followed by a 37-degree water bath for 1 h, and centrifugation at 1500 g for 5 min. Add the supernatant to the 96-well plate, and test the absorbance of each group (OD: 570 nm).

### Cell Culture

Treg cells were isolated from peripheral blood of pig receptors using EasySep™ CD4^+^CD127^low^CD25^+^ Treg sorting kit (Novobiotec) and cultured in RPMI1640 containing 10% fetal bovine serum (Hyclone). Treg cells were further expanded to 3×10^9^ with the Treg Expansion Kit (Novobiotec). Isolation and culture of EPCs were performed using the method previously reported. In short, peripheral blood from diabetic patients was collected and mononuclear cells were isolated using lymphocyte separation fluid (Tianjin Haoyang Biological Manufacture Co., Ltd). Isolated mononuclear cells were cultured in an EGM-2 complete medium (Lonza) containing 15% fetal bovine serum. After 48h, unattached cells were removed, and the attached cells continued selective culture.

### Animal Experiment

All animal experiments were performed in accordance with the regulations on animal experiments of Chinese PLA General Hospital (Beijing, China) and were approved by the Ethics Committee of Chinese PLA General Hospital (NO: 309201803051058). Referring to previous reports (Lewczuk et al., 2018; Pierzynowska et al., 2020), we used streptozotocin (Sigma) injection to construct a diabetic pig model. After fasting for 16 h, pigs weighing about 60 kg were anesthetized with ketamine (35 mg/kg) and diazepam (1.5 mg/kg), and streptozotocin (150 mg/kg) injected *via* ear vein. Fasting blood glucose was measured 1, 2, 3, and 7 days after administration. Fasting blood glucose > 7 mmol for 7 consecutive days was considered the successful establishment of the diabetes model.

After anesthesia, the common carotid artery was carefully separated after routine disinfection. The proximal and distal ends were blocked with arterial clamps. After the carotid artery was severed, anti-CD34 antibody modified TEBV (about 2.5 cm in length) was anastomosed. Stop the bleeding carefully and close the skin layer by layer. Aspirin enteric-coated tablets (0.1 g/day, orally) and low molecular weight heparin calcium injection (5, 000IU/day, subcutaneously) were given postoperatively. The experiment was divided into four groups: scaffold group (implanting vascular scaffold coated with heparin-collagen multilayer), control group (implanting anti-CD34 antibody modified TEBV), peripheral injection group (Treg cells were injected around TEBV after implantation of anti-CD34 antibody modified TEBV) and intravenous injection group (Treg cells were injected *via* ear vein after implantation of anti-CD34 antibody modified TEBV). There were 8 animals in each group.

### Specimen Sampling and Histological Staining

After 60 days of implantation, animals in each group were anesthetized. The implanted TEBVs were collected and fixed in 10% paraformaldehyde for 24 h. After paraffin sections, some sections were stained with hematoxylin and eosin (H&E, Sigma) and some were immunohistochemistry. In short, paraffin sections were routinely dewaxed to water, treated with 3% H_2_O_2_ for 5 min, and then sealed with sheep serum working solution for 5min anti-CD31, SM-actin, ki-67, CD68, LCA, CD4, CD8, CD20, CD56, CD38 primary antibodies (All purchased from Abcam) were added and incubated overnight at 4°. After washing with PBS 3 times, a biotin-labeled secondary antibody was added and incubated at 37° for 30 min, then washed with PBS 3 times. Streptomycin working solution labeled with horseradish enzyme was added and washed with PBS 3 times, and the color was displayed for 10 min. Rinse thoroughly with running water, redye with hematoxylin, and seal sheet. Both Nestin^+^ cells and CD90^+^ cells showed stem cell characteristics and had the potential to differentiate into endothelial cells or smooth muscle cells after implantation of bioartificial blood vessels (Kirkton et al., 2019). Therefore, we further detected the number of Nestin^+^ cells and CD90^+^ cells in the implanted TEBVs by immunohistochemistry. Under high power field (HPF), 10 fields were randomly selected to count positive staining cells in each section, and then the average value was calculated.

### TUNEL Staining

The slices were treated with 3% H_2_O_2_ for 10 min and added with 0.1% Triton X-100 for 2 min. After washing with PBS 3 times, the reaction mixture (Roche) was added and incubated at 37° for 60 min. After that, DAPI (Sigma) was added to stain the nuclei and seal the sheet after washing with PBS 3 times.

### Flow Cytometry

Peripheral blood of each group was collected 7 days after TEBV implantation. Mononuclear cells were isolated by a lymphocyte separation medium. After washing with PBS 3 times, the fluorescent-dye conjuncted antibodies of CD34, VEGFR-2, ICAM-1, VCAM-1, and E-selection (All purchased from BD) were added and incubated in dark for 15min. After washing with PBS 3 times, the proportion of EPCs and mean fluorescence intensity (MFI) of ICAM-1, VCAM-1, and E-selection was detected by flow cytometry (BD FACSCanto flow cytometer). Plasma was also collected and the concentrations of VEGF and SDF-1 were detected by ELISA (Huijia). The operation was carried out strictly in accordance with ELISA kit instructions.

### Co-Culture Experiment

A Transwell chamber was used for the cell co-culture experiment, Treg cells were added into the upper chamber, and mononuclear cells isolated from peripheral blood of diabetic patients were added into the lower chamber. The experiment was divided into 6 groups: diabetes group (untreated mononuclear cells), Treg cell group (Treg cells were co-cultured with mononuclear cells), IL-10 Neu group (Treg cells were co-cultured with mononuclear cells and IL-10 neutralizing antibody were added), TGF-β Neu group (Treg cells were co-cultured with mononuclear cells and TGF-β neutralizing antibody were added), HLA-G Neu group (Treg cells were co-cultured with mononuclear cells and HLA-G neutralizing antibody were added), and CTLA-4 Neu group (Treg cells were co-cultured with mononuclear cells and CTLA-4 neutralizing antibody were added). After co-culture for 48 h, cells of each group were collected. After washing with PBS 3 times, CD34-PE and VEGFR-2-APC (All purchased from BD) were added and incubated in dark for 15min. After washing with PBS 3 times, the proportion of EPCs was detected by flow cytometry.

We further conducted the co-culture experiment of Treg cells and EPCs. After 48 h of co-culture, Treg cells in the upper chamber were removed. After continued culture for 48 h, the supernatant was collected and the concentrations of VEGF and SDF-1 were detected by ELISA. The operation was carried out strictly in accordance with ELISA kit instructions.

EPCs of each group were collected after 1 week. After washing with PBS 3 times, CD31-PE (BD) was added and incubated for 15 min. After washing with PBS 3 times, the proportion of CD31^+^cells were detected by flow cytometry.

### Statistical Evaluation

Data are presented as mean ± SEM. GraphPad Software 5.0 was used for all statistical calculations. To ascertain the significance of differences, we performed ANOVA and Bonferroni post hoc test. A value of *p* < 0.05 was considered statistically significant.

## Results

### Characteristics of the Anti-CD34 Antibody-Modified Tissue-Engineered Blood Vessel

**FIGURE 1:**
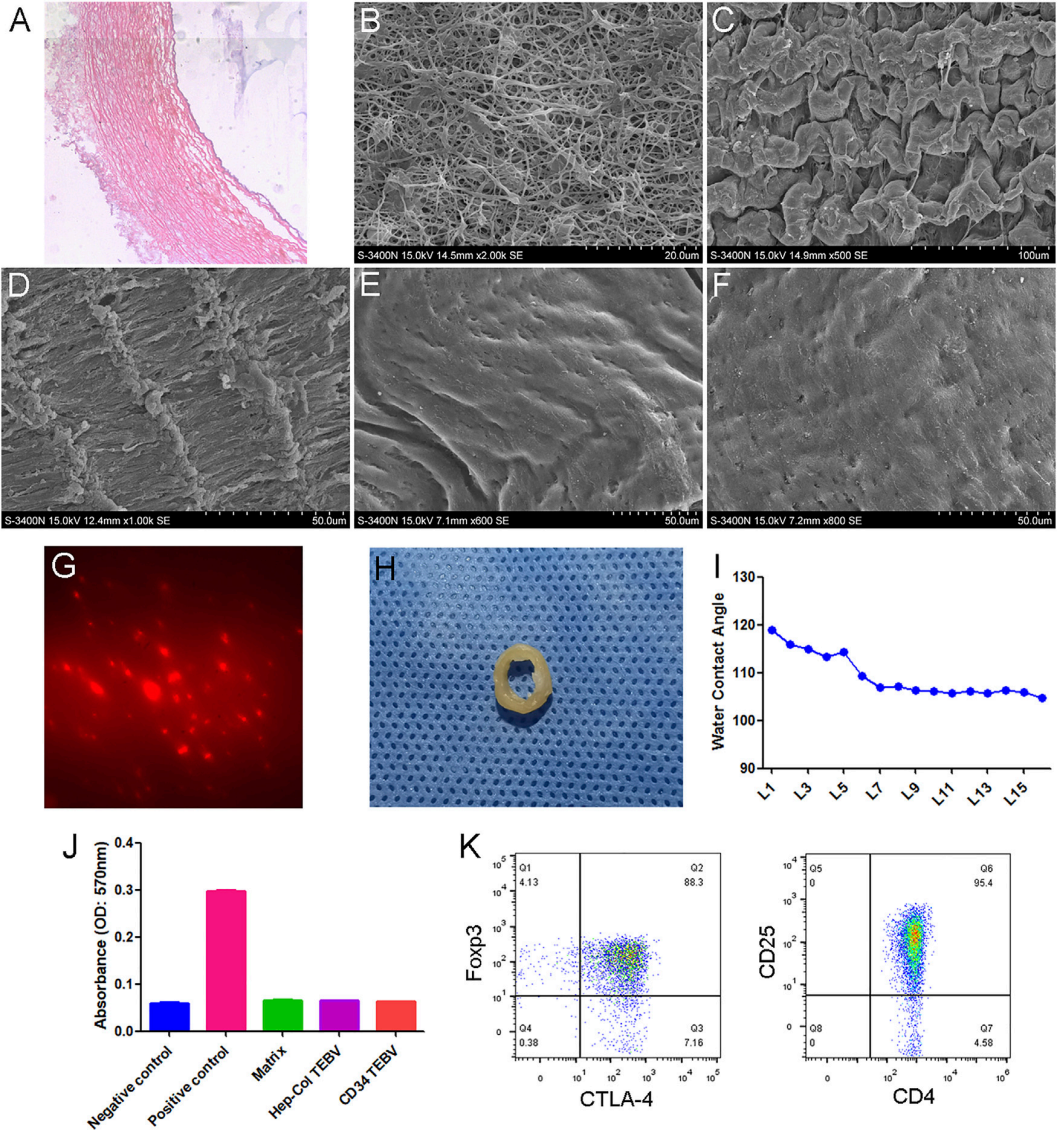
Characteristics of anti-CD34 antibody modified TEBV. **(A)** H&E staining of TEBV acellular scaffold. **(B)** Transmission electron microscopy images. Scanning electron microscope showing the surface of TEBV scaffold before coating **(C)** and after coating 2 layers **(D)**, 5 layers **(E),** and 10 layers **(F)**. **(G)** Red fluorescence (CD34-PE) could be observed on the surface of TEBV after 2 h of washing with normal saline. **(H)** The general picture of anti-CD34 antibody modified TEBV. **(I)** Water Contact Angle test results showed that the contact angle decreased with the increase of coating layers. The contact angle did not change significantly after 7 layers of coating. **(J)** *In vitro* hemolysis experiments showed that coating of collagen, heparin and CD34 antibody had no significant effect on the hemolysis of the acellular vascular matrix. **(K)** Flow cytometry results showed that more than 90% of the expanded cells expressed both CD4 and CD25. Foxp3 and CTLA-4 were also highly expressed.

H&E staining and transmission electron microscopy showed that the TEBV scaffold was basically composed of fibrous tissue, without any cells (Figures 1A,B). A scanning electron microscope showed that the surface of the TEBV scaffold was flatter and smoother with the increase of coating layers (Figures 1C–F). Figure 1G showed that red fluorescence (CD34-PE) could be observed on the surface of TEBV after 2 h of washing with normal saline. Water Contact Angle test results showed that the contact angle decreased with the increase of coating layers. The contact angle did not change significantly after 7 layers of coating (Figure 1I). *In vitro* hemolysis experiments showed that the coating of collagen, heparin and CD34 antibody had no significant effect on the hemolysis of the acellular vascular matrix (Figure 1J). Treg cells were sorted and expanded *in vitro*. Flow cytometry results showed that more than 90% of the expanded cells expressed both CD4 and CD25, and more than 85% expressed both Foxp3 and CTLA-4 (Figure 1K).

Treg cell transplantation inhibited intimal hyperplasia of anti-CD34 antibody modified TEBV in diabetes mellitus.

**FIGURE 2:**
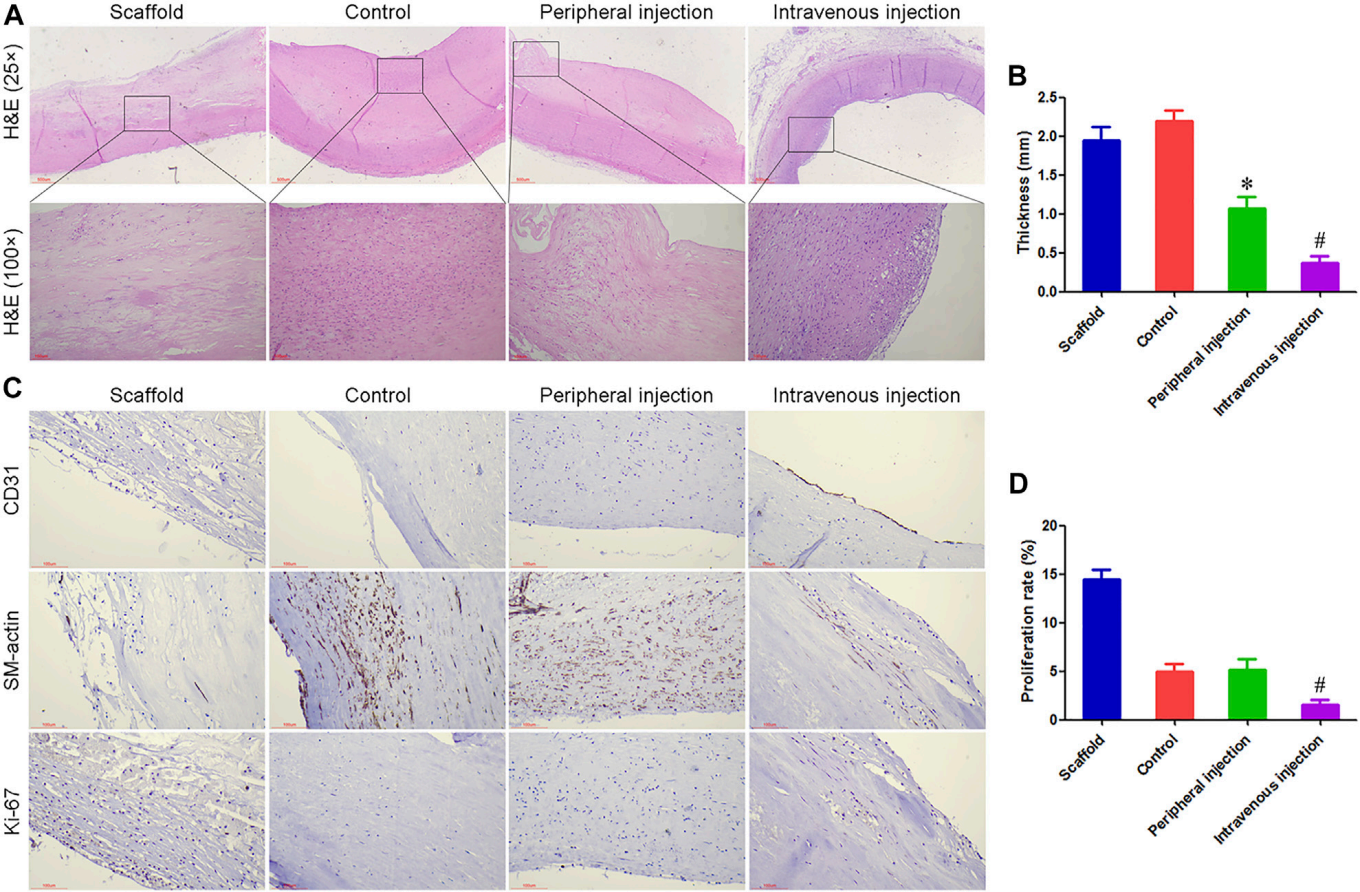
Treg cell transplantation inhibited intimal hyperplasia of anti-CD34 antibody modified TEBV in diabetes mellitus. **(A)** H and E staining, all TEBV were not completely blocked at 60 days after implantation. **(B)** The thickness of intimal hyperplasia in the intravenous injection group was smaller than that in the control group and peripheral injection group. **(C)** Immunohistochemistry was used to detect the expression of CD31, SM-actin, and ki-67. An intact endothelial cell layer was observed in the intravenous injection group. **(D)** Ki-67 proliferation index in the intravenous injection group was smaller than that in the control group and peripheral injection group. *p < 0.05 (*n* = 8) versus Control group. ^#^ *p* < 0.05 (*n* = 8) versus Peripheral injection group. Values are mean ± SD.

At 60 days after implantation, all TEBV samples were not completely blocked, mainly due to the heparin coating and anticoagulant therapy. The inner surface of TEBV in the scaffold group was mainly composed of thrombus and organized thrombus, and severe damage and degradation of the TEBV matrix were observed. If further degradation *in vivo*, TEBVs rupture, and massive bleeding may occur. Different degrees of intimal hyperplasia were observed in the control group, peripheral injection group, and intravenous injection group, which did not have continuity with the normal smooth muscle layer in the TEBV scaffold, suggesting that smooth muscle cells in the intimal hyperplasia may be mainly derived from the differentiation of peripheral blood stem cells. The thickness of intimal hyperplasia in the peripheral injection group was smaller than that in the control group, but there was no statistical difference in the ki-67 proliferation index. The thickness of intimal hyperplasia was the smallest in the intravenous injection group, and an intact endothelial cell layer was observed. Figure 2.

Treg cell transplantation reduced the inflammatory response associated with TEBV implantation in diabetes mellitus.

**FIGURE 3:**
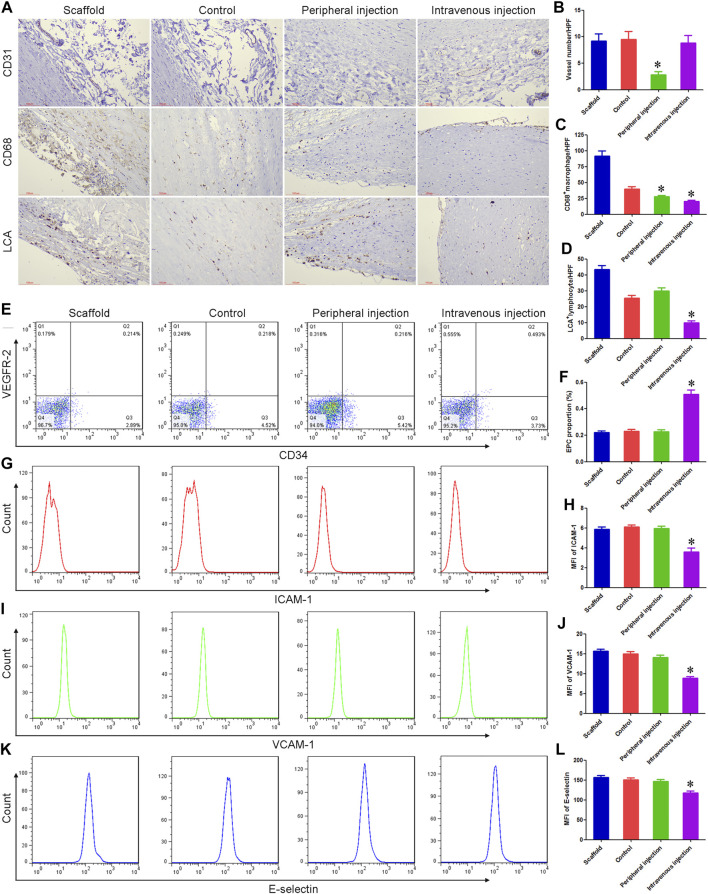
Treg cell transplantation reduced the inflammatory response associated with TEBV implantation in diabetes mellitus. **(A)** Immunohistochemistry was used to detect the expression of CD31, CD68, and lymphocyte common antigen (LCA). **(B–D)** Compared with the control group, the number of macrophages (CD68^+^) and microvessel density (CD31^+^) around TEBV decreased in the peripheral injection group, respectively, while the number of lymphocytes (LCA^+^) did not differ statistically between the two groups. Compared with the control group, there was no significant change in CD31^+^ microvessel density around TEBV in the intravenous injection group, while the number of lymphocytes and macrophages was significantly reduced. **(E)** Flow cytometry was used to detect the proportion of EPCs in peripheral blood. **(F)** Compared with the control group and peripheral injection group, the proportion of EPCs was significantly increased in the intravenous injection group. **(G,I,K)** Flow cytometry was used to detect the expression of ICAM-1, VCAM-1, and e-selectin on EPCs. **(H,J,L)** Compared with the control group and peripheral injection group, the expression of ICAM-1, VCAM-1, and e-selectin was significantly decreased in the intravenous injection group. *p < 0.05 (*n* = 8) versus Control group. Values are mean ± SD.

Immunohistochemistry was used to label the number of lymphocytes and macrophages, and the density of microvessels. A large number of macrophages and lymphocytes were observed in the scaffold group, which may be an important reason for the rapid degradation of vascular matrix materials. Compared with the control group, the number of macrophages and CD31^+^ microvessel density around TEBV decreased by 30.53% and 70.18% in the peripheral injection group, respectively, while the number of lymphocytes did not differ statistically between the two groups. Compared with the control group, there was no significant change in CD31^+^ microvessel density around TEBV in the intravenous injection group, while the number of lymphocytes and macrophages was significantly reduced. Figures 3A–D.

Peripheral blood of each group was collected 7 days after TEBV implantation, and the proportion of EPCs and the expression of pro-inflammatory molecules were detected. Flow cytometry showed no statistical difference in the proportion of peripheral blood EPCs and the expression of ICAM-1, VCAM-1, and E-selectin between the scaffold group, control group, and peripheral injection group. ELISA results showed that there were no significant differences in plasma VEGF and SDF-1 concentrations among the three groups. Compared with the control group, the proportion of EPCs and the concentration of VEGF and SDF-1 were significantly increased in the intravenous injection group, while the expression of ICAM-1, VCAM-1, and e-selectin was significantly decreased. These results suggest that Treg cells can inhibit angiogenesis in the ischemic microenvironment (around TEBV), possibly by inhibiting the vascularization process initiated by an inflammatory response. On the other hand, Treg cells can promote angiogenesis and rapid endothelialization in nutrient and oxygen-rich sites (in TEBV) by enhancing mobilization and paracrine of EPCs and decreasing the expression of pro-inflammatory molecules. Figures 3E–3L and Supplementary Figures S1, S2.

We further detected the number of different lymphocyte subsets in TEBV. Immunohistochemical results showed that there was no statistical difference in the number of CD4^+^T cells, CD8^+^T cells, B cells, plasma cells, and NK cells between the scaffold group, control group, and peripheral injection group. Compared with the control group, the number of CD8^+^T cells was significantly reduced in the intravenous injection group, while there were no significant differences in CD4^+^T cells, B cells, plasma cells, and NK cells. These results suggest that CD8^+^T cells may play a more important role after TEBV implantation Supplementary Figure S3.

Treg cell transplantation inhibited cell apoptosis in anti-CD34 antibody-modified TEBV.

**FIGURE 4:**
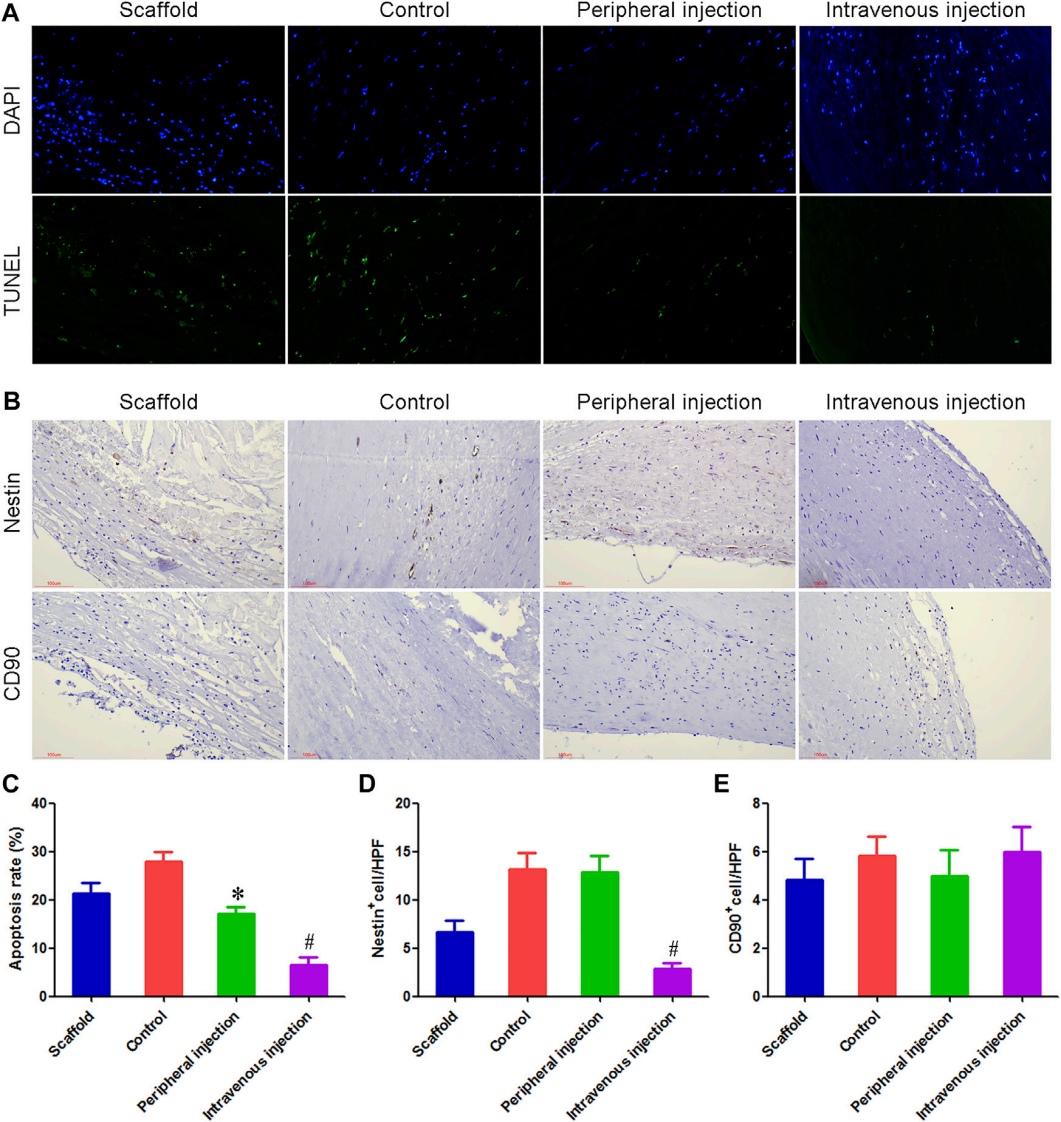
Treg cells intravenous transplantation reduced the number of apoptotic cells and Nestin + cells in anti-CD34 antibody modified TEBV. **(A)** TUNEL staining was used to detect cell apoptosis. **(B)** Immunohistochemistry was used to detect the number of Nestin^+^ cells and CD90^+^ cells. **(C)** Compared with the control group and peripheral injection group, the number of apoptotic cells was significantly decreased in the intravenous injection group. **(D)** Immunohistochemical results showed that many Nestin^+^ cells could be detected in both the control group and peripheral injection group, most of which were fusiform, showing the characteristics of smooth muscle cells. Compared with the control group and peripheral group, the number of Nestin^+^ cells were reduced by 78.51% and 77.92% in the intravenous injection group, respectively. **(E)** There was no statistical difference in the number of CD90^+^ cells among the four groups. *p < 0.05 (*n* = 8) versus Control group. ^#^ *p* < 0.05 (*n* = 8) versus Peripheral injection group. Values are mean ± SD.

TUNEL staining results showed that the percentage of apoptotic cells in the control group and peripheral injection group was 27.83% and 17.17%, respectively, and the difference was statistically significant. The percentage of apoptotic cells in the intravenous injection group was 6.58%, and the differences were statistically significant compared with the control group and peripheral injection group. Figures 4A,C.

Treg cells intravenous transplantation reduced the number of Nestin^+^ cells in anti-CD34 antibody modified TEBV.

Immunohistochemical results showed that many Nestin^+^ cells could be detected in both the control group and peripheral injection group, most of which were fusiform, showing the characteristics of smooth muscle cells. This result indicated that in diabetes, Nestin^+^ cells mainly differentiated into vascular smooth muscle cells and promoted the occurrence of intimal hyperplasia. Compared with the control group and peripheral group, Nestin^+^ cells were reduced by 78.51% and 77.92% in the intravenous injection group, respectively. There was no statistical difference in the number of CD90^+^ cells among the four groups. Figures 4B,D,E.

Effect of Treg cells on EPCs function in diabetic patients.

**FIGURE 5:**
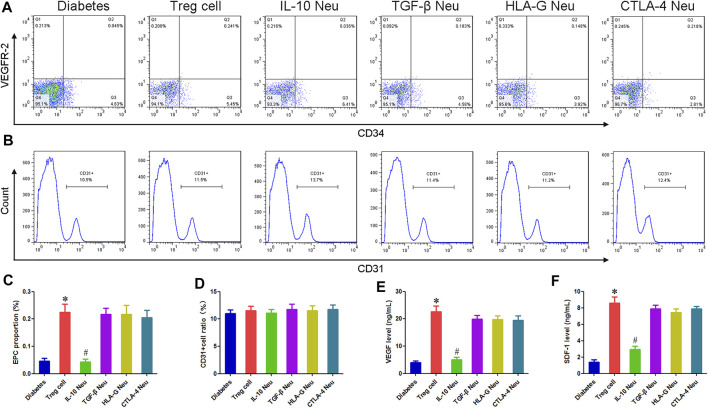
Effect of Treg cells on EPCs function in diabetic patients. **(A,B)** Flow cytometry was used to detect the proportion of EPCs and CD31^+^cells. **(C)** Compared with the diabetes group, the proportion of EPC in the Treg cell group increased markedly. IL-10 neutralization could inhibit this effect on Treg cells. **(D)** There was no statistical difference in the proportion of CD31^+^ endothelial cells among all groups. **(E,F)** Compared with the diabetes group, the concentration of VEGF and SDF-1 in the EPC supernatant of the Treg cell group increased by 4.75 and 5.13 times, respectively. IL-10 neutralization can inhibit these effects of Treg cells. *p < 0.05 (*n* = 6) versus Diabetes group. ^#^ *p* < 0.05 (*n* = 6) versus Treg cell group. Values are mean ± SD.

Compared with the diabetes group, the proportion of EPC in the Treg cell group increased markedly, indicating that Treg cells can promote the proliferation of EPC in diabetes mellitus. IL-10 neutralization could inhibit this effect of Treg cells, and the EPC proportion in the IL-10 Neu group was reduced by 80.89% compared with the Treg cell group. The neutralization of TGF-β, CTLA-4, and HLA-G had no significant effect. Figures 5A,C.

Compared with the diabetes group, the concentration of VEGF and SDF-1 in the EPC supernatant of the Treg cell group increased by 4.75 and 5.13 times, respectively. IL-10 neutralization can inhibit these effects of Treg cells, and the concentration of VEGF and SDF-1 in the IL-10 Neu group decreased by 77.73% and 65.63%, respectively, compared with the Treg cell group. The neutralization of TGF-β, CTLA-4, and HLA-G had no significant effect. Figures 5E,F.

After 1 week of co-culture, we detected the proportion of CD31^+^ endothelial cells. Flow cytometry results showed that there was no statistical difference in the proportion of CD31^+^ endothelial cells among all groups, indicating that Treg cells had no significant effect on the differentiation of EPCs. Figures 5B,D.

## Discussion

EPC captured TEBV is considered the development direction of the new generation of TEBV, which can maintain a good patency effect in animal experiments (Hao et al., 2020). However, many patients with cardiovascular and cerebrovascular diseases are often accompanied by basic diseases such as diabetes. The microenvironment of high glucose and high oxidative stress damages the function of homing EPCs and promotes the pathological proliferation of vascular smooth muscle cells, leading to the implantation failure of TEBV (Chen et al., 2018). Our previous study showed that the patency rate of EPC-captured TEBV was very low in diabetic rats. In this study, we constructed a diabetic pig model and found that all implanted TEBVs were not completely blocked, mainly due to heparin coating and anticoagulant therapy. However, 60 days after implantation, intimal hyperplasia occurred in the control TEBV, while intravenous injection of Treg cells significantly inhibited intimal hyperplasia. Immunohistochemical results showed that the number of macrophages, CD8^+^T cells, and apoptotic cells were significantly reduced in the intravenous injection group. Interestingly, our study also showed that Treg cells decreased the number of Nestin^+^ cells in TEBV. Clinical results showed that Nestin^+^ cells and CD90^+^ cells simultaneously expressed CD44, CD73, and CD140a, and had the potential to differentiate into endothelial cells or smooth muscle cells after implantation of bioartificial blood vessels (Kirkton et al., 2019). Our results indicated that in diabetes, Nestin^+^ cells mainly differentiated into vascular smooth muscle cells and promoted the occurrence of intimal hyperplasia.

Successful implantation of biomaterials *in vivo* is closely related to the inflammatory response. Low intensity inflammatory response can induce homing of EPCs and initiate the vascularization process of biomaterials (Garg et al., 2008). However, the over-activation of inflammatory response in diabetes mellitus will further damage the function of homing EPCs and inhibit angiogenesis in biomaterials. At present, the correlation between Treg cells and angiogenesis is highly controversial. Some studies have found that Treg cells can inhibit angiogenesis in ischemic tissues, anti-CD25 treatment, and subsequent Treg deletion were significantly enhanced postischemic neovasculplasmin (Zouggari et al., 2009). But other studies found that Treg cells were the main source of VEGF, which can induce angiogenesis. After TEBV implantation, the peripheral blood circulating in the lumen contains sufficient oxygen and nutrients, while the damaged tissues around TEBV are relatively ischemic and hypoxia, so it can be used as an important model to study the relationship between Treg cells and angiogenesis. In order to sufficiently isolate the exchange of matter between the inside and outside of the TEBV, we transplanted TEBV into a pig model. The results showed that intravenous injection of Treg cells could promote long-term patency of TEBV and inhibit intimal hyperplasia, while Treg cells used around TEBV had no significant effect, and microvessel density around TEBV was significantly reduced. After implantation *in vivo*, the surrounding tissues of TEBV are a relatively ischemic and anoxic microenvironment, and inflammatory cells in the blood arrive at a very late time and are difficult to survive. Treg cells further inhibited the activation and function of inflammatory cells, thus weakening angiogenesis.

Treg cells exert immunosuppressive function mainly through two mechanisms. Treg cells highly express HLA-G and CTLA-4, which can inhibit the proliferation and activation of immune cells through direct contact or exosome (Hao et al., 2007; Zhang et al., 2018; Rana and Biswas, 2020). Treg cells can also secrete IL-10 and TGF-β, indirectly exerting biological functions (Song et al., 2021; Tagkareli et al., 2022). Clinical studies have shown that Treg cells are positively correlated with early EPCs, but the specific mechanism is unclear. Our results showed that intravenous injection of Treg cells after TEBV implantation could improve the proportion of EPCs and the concentration of VEGF in peripheral blood. *In vitro* experiments further confirmed that IL-10 mediated the angiogenic function of Treg cells. EPCs also highly produce IL-10, TGF-β, and HLA-G, these cytokines in EPCs will be the potential pathways in immune regulation (Naserian, 2020). Recent articles demonstrated that TNFR1 and Tie-2 can be used as the important markers for tissue-engineered materials related to inflammation detection (Nouri Barkestani et al., 2021). Some studies have shown that an important target of IL-10 action is STAT3, which promotes the expression and secretion of VEGF and SDF-1 (Wang et al., 2015; Shirakawa et al., 2018). Therefore, we believe that Treg cells in the ischemic tissues (around the TEBV) mainly block the initiation of vascularization by inhibiting the infiltration and function of inflammatory cells, while Treg cells in the tissue with abundant blood circulation (in the TEBV) mainly promote the mobilization of EPCs and the secretion of angiogenic factors through the IL-10 pathway.

In conclusion, Intravenous transplantation of Treg cells can delay the occurrence of intimal hyperplasia in diabetic pigs by promoting EPCs mobilization, anti-inflammatory action, and cellular protection. We also found that the correlation between Treg cells and angiogenesis was mainly closely related to the microenvironment. Our study provides a new target for the clinical transformation of TEBV and provides new theoretical support for understanding the correlation between inflammation and angiogenesis.

## Data Availability

The original contributions presented in the study are included in the article/Supplementary Material; further inquiries can be directed to the corresponding authors.
