# Supplementary material for: The Inhibitory Effect of Regulatory T Cells on the Intimal Hyperplasia of Tissue-Engineered Blood Vessels in Diabetic Pigs
Source: Front Bioeng Biotechnol. 2022 Jul 26;10:929867. doi: 10.3389/fbioe.2022.929867 (PMC9360552; doi:10.3389/fbioe.2022.929867)
Supplement: Supplementary file 1 [file DataSheet1.docx]

**The inhibitory effect of regulatory T cells on the intimal hyperplasia of tissue engineered blood vessels in diabetic pigs**

Fengjie Guo ^a^, Zhipeng Ren ^b^, Dongxu Liu ^a^, Linghui Wang ^b^, Xiaobin Hou ^b^, Wen Chen ^a^

a. Department of Pathology, The 8th Medical Center of Chinese PLA General Hospital, Beijing 100091, China

b. Department of thoracic surgery, The First Medical Center, Chinese PLA General Hospital, Beijing 100853, China.

Corresponding author: Wen Chen, E-mail: dr.chen20160224@foxmail.com; Xiaobin Hou, E-mail: drhouxb@163.com


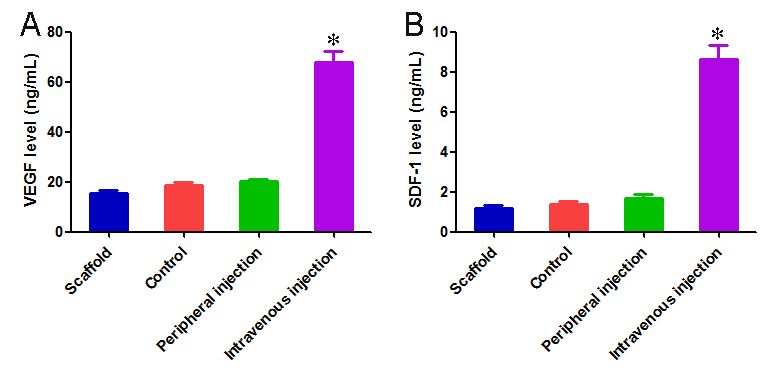
Figure S1. ELISA results showed that there were no significant differences in plasma VEGF and SDF-1 concentrations among the scaffold group, control group and peripheral injection group. Compared with the control group, the proportion of EPCs and the concentration of VEGF and SDF-1 were significantly increased in the intravenous injection group. **p*<0.05 (n=8) versus Control group. Values are mean ± SD.


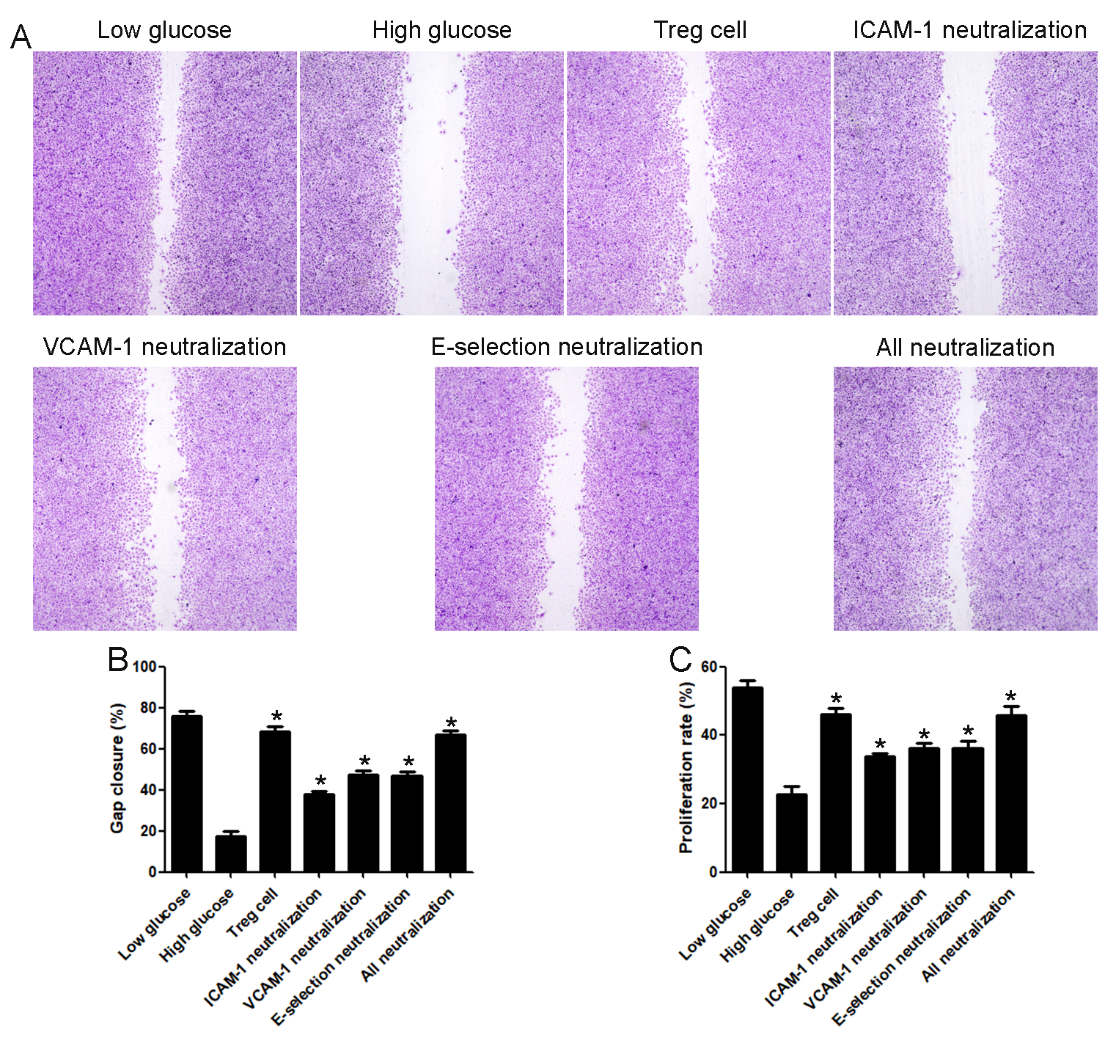


Figure S2. EPCs were divided into seven groups: Low glucose group (5.6 mM glucose), high glucose group (30 mM glucose), Treg cell group (Treg cells were added in high glucose medium), ICAM-1 neutralization group (ICAM-1 neutralizing antibody was added in high glucose medium), VCAM-1 neutralization group (VCAM-1 neutralizing antibody was added in high glucose medium), E-selectin neutralization group (E-selectin neutralizing antibody was added in high glucose medium) and All neutralization group (ICAM-1, VCAM-1 and E-selectin neutralizing antibody were added in high glucose medium). After 48h of stimulation, EPCs were washed with PBS for three times, and co-cultured with monocytes/macrophages. After 24h, monocytes/macrophages in each group were collected and co-cultured with endothelial cells. Wound healing assay and MTT experiments showed that under high glucose conditions, Treg cells promoted the migration and proliferation of endothelial cells by inhibiting the activation of EPCs on monocytes/macrophages. The neutralization of ICAM-1, VCAM-1 or E-selectin can partially promote the migration and proliferation of endothelial cells.


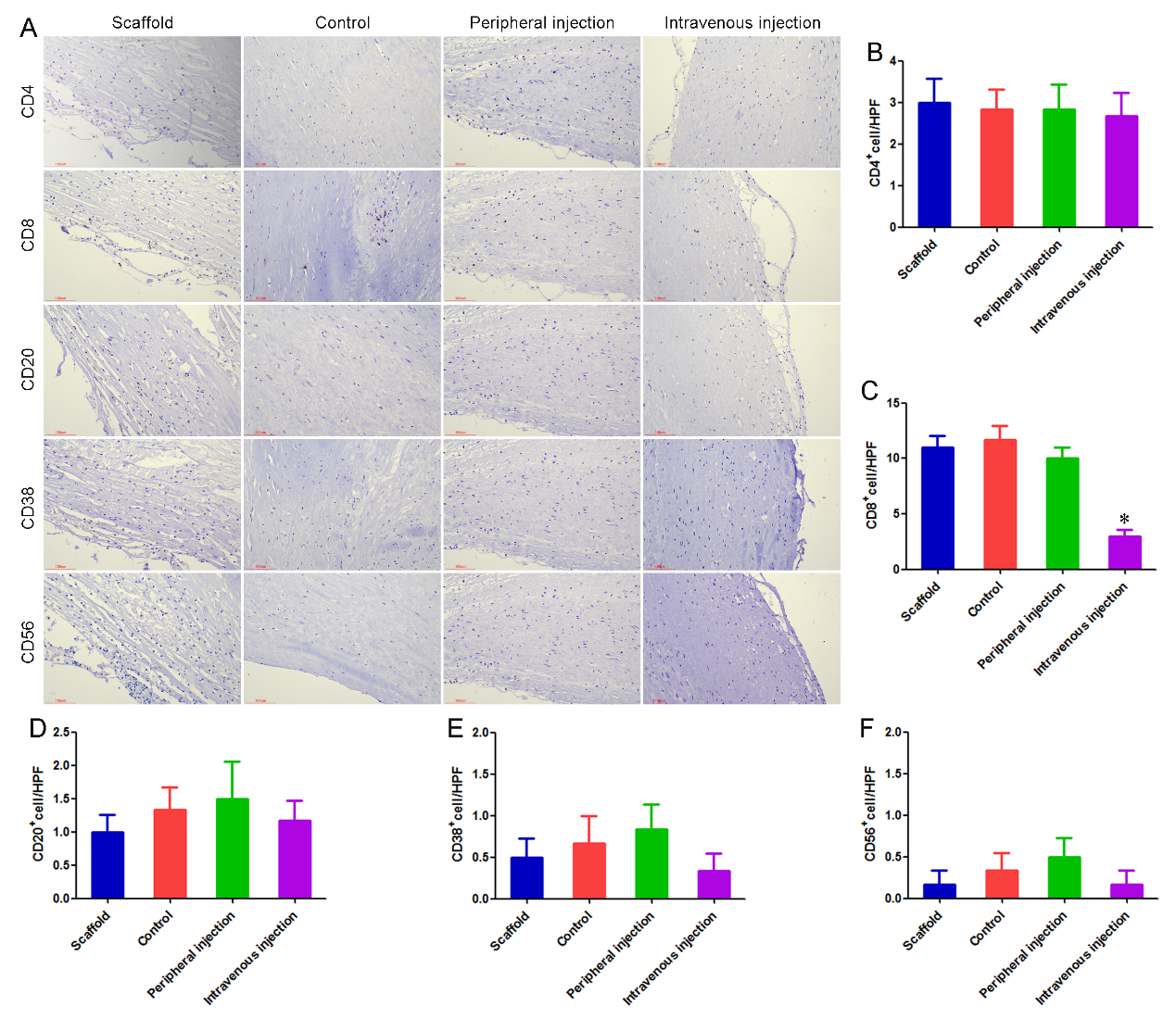


Figure S3. The number of different lymphocyte subsets in TEBV was detected by immunohistochemistry. Results showed that there was no statistical difference in the number of CD4^+^T cells, CD8^+^T cells, B cells, plasma cells and NK cells between the scaffold group, control group and peripheral injection group. Compared with the control group, the number of CD8^+^T cells was significantly reduced in the intravenous injection group, while there were no significant differences in CD4^+^T cells, B cells, plasma cells, and NK cells. These results suggest that CD8^+^T cells may play a more important role after TEBV implantation. **p*<0.05 (n=8) versus Control group. Values are mean ± SD.
